# A Giant Dumbbell Shaped Vesico-Prostatic Urethral Calculus: A Case Report and Review of Literature

**DOI:** 10.1155/2013/167635

**Published:** 2013-05-21

**Authors:** Vinod Kumar Prabhuswamy, Rahul Tiwari, Ramakrishnan Krishnamoorthy

**Affiliations:** Department of General Surgery, Victoria Hospital, Bangalore Medical College and Research Institute, Bangalore 560002, India

## Abstract

Calculi in the urethra are an uncommon entity. Giant calculi in prostatic urethra are extremely rare. The decision about treatment strategy of calculi depends upon the size, shape, and position of the calculus and the status of the urethra. If the stone is large and immovable, it may be extracted via the perineal or the suprapubic approach. In most of the previous reported cases, giant calculi were extracted via the transvesical approach and external urethrotomy. A 38-year-old male patient presented with complaints of lower urinary tract symptoms. Further investigations showed a giant urethral calculus secondary to stricture of bulbo-membranous part of the urethra. Surgical removal of calculus was done via transvesical approach. Two calculi were found and extracted. One was a huge dumbbell calculus and the other was a smaller round calculus. This case was reported because of the rare size and the dumbbell nature of the stone. Giant urethral calculi are better managed by open surgery.

## 1. Introduction

Urinary calculi are the third most common affliction of the urinary tract, exceeded only by urinary tract infections and pathologic conditions of the prostate [1]. Urethral calculi represent 1-2% of all calculi in the genito-urinary tract [2]. Primary urethral calculi are usually associated with urethral strictures, posterior urethral valve, or a diverticula [3]. Giant calculi occurring in prostatic urethra are extremely rare. Only few cases of giant prostatic urethral calculi are reported in literature. Here we present a case of giant dumbbell shaped calculus in prostatic urethra. 

## 2. History and Clinical Examination

A 38-year-old male patient, labourer by occupation came with complaints of decreased urinary stream, straining during micturition, and dysuria for six months. He had no history of haematuria, fever, vomiting, or trauma, but the patient had feeling of incomplete emptying. He was evaluated for these symptoms in a local hospital where X ray KUB and urethra were done and then he was referred to our hospital for further management. The patient had a significant past history of undergoing a suprapubic catheterisation and some endoscopic urethral procedure but without any documents. Clinical examination of abdomen revealed a scar in the suprapubic region.

Calculus was palpable in the perianal region and also on per rectal examination. External genitalia was normal.

## 3. Investigations

**Figure 1:**
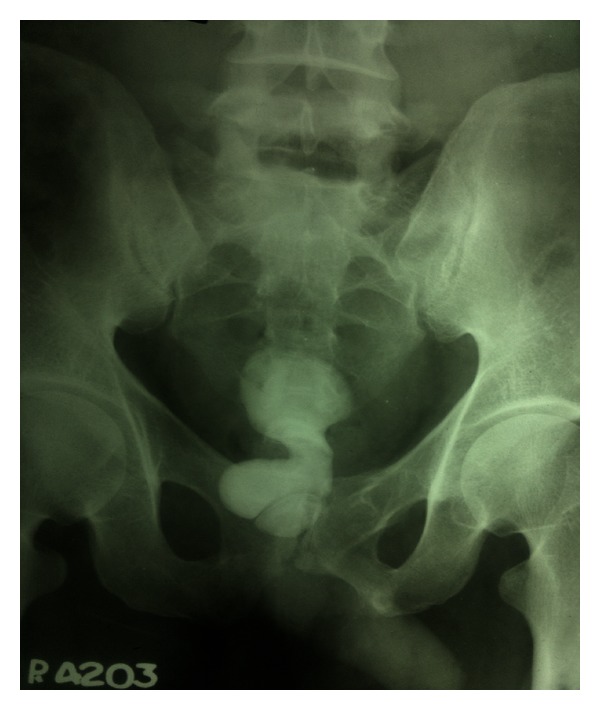
Erect X-ray KUB showing a giant calculus in the lower urinary tract.

**Figure 2:**
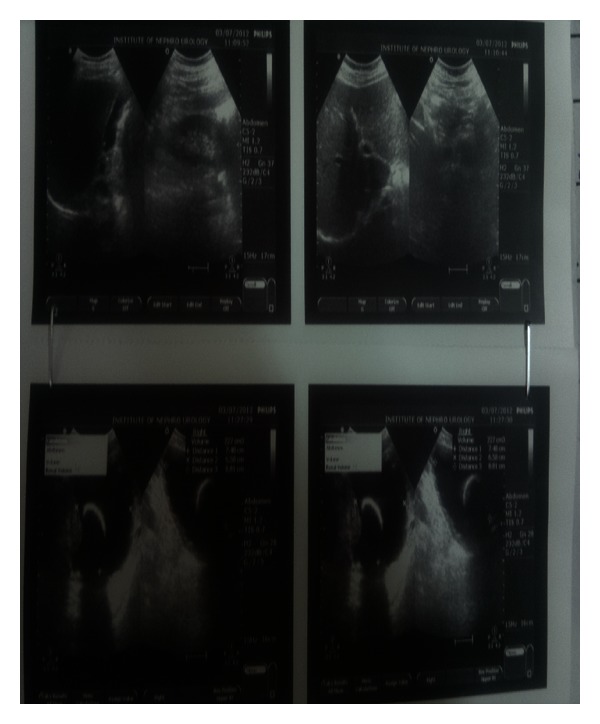
Ultrasonogram showing the giant calculus in bladder extending into urethra.

**Figure 3:**
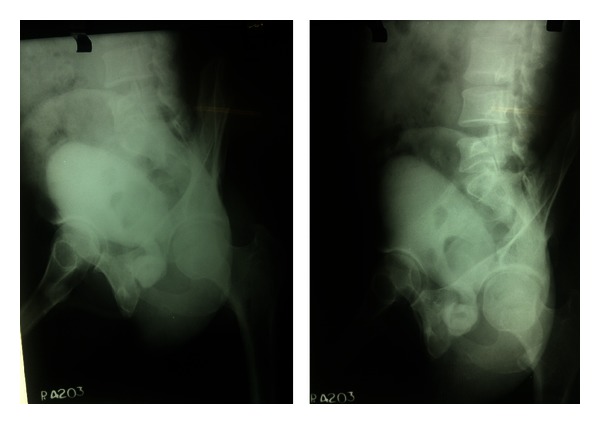
Micturating cystourethrogram showing stricture in the bulbar urethra and a calculus in proximal prostatic urethra.

**Figure 4:**
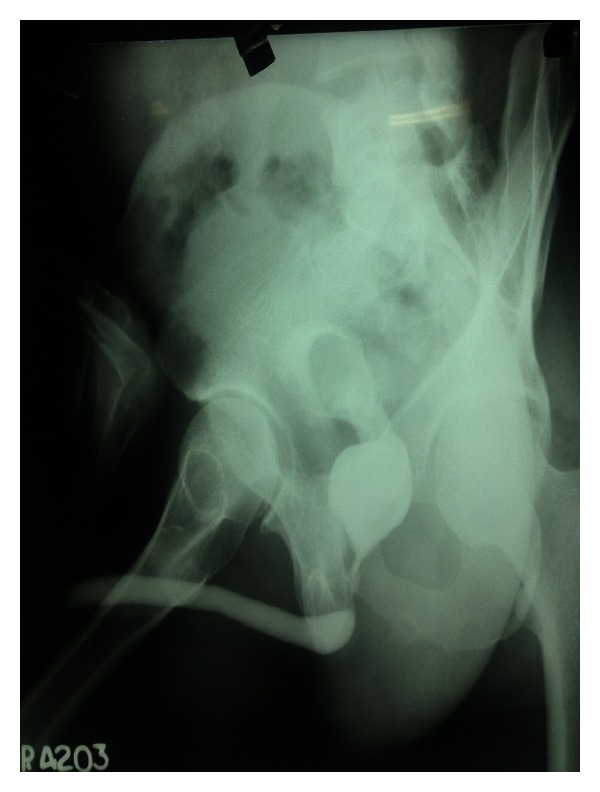
Retrograde cystourethrogram showing stricture of bulbomembranous junction with dilated prostatic urethra with calculus.

 Haematology: complete blood count, renal function tests, liver function tests, serum electrolytes, serum parathyroid hormone, and serum calcium levels were within normal limits.
 Urine Routine and Microscopy: traces of albumin & 3-4 RBCs and plenty of pus cells/HPF.
 Urine culture grew *Escherichia*.
 Plain X-Ray KUB was repeated here which showed a huge calculus, dumbbell shaped in the region of bladder and prostatic urethra (Figure 1).
 Ultrasound abdomen revealed bilateral mild hydro-ureteronephrosis and thickening of the bladder wall (Figure 2).
 Micturating cystourethrography shows stricture in the bulbar urethra and a calculus in proximal prostatic urethra (Figure 3).
 Retrograde cystourethrography stricture of bulbo-membranous junction with dilated prostatic urethra with calculus (Figure 4).

**Figure 5:**
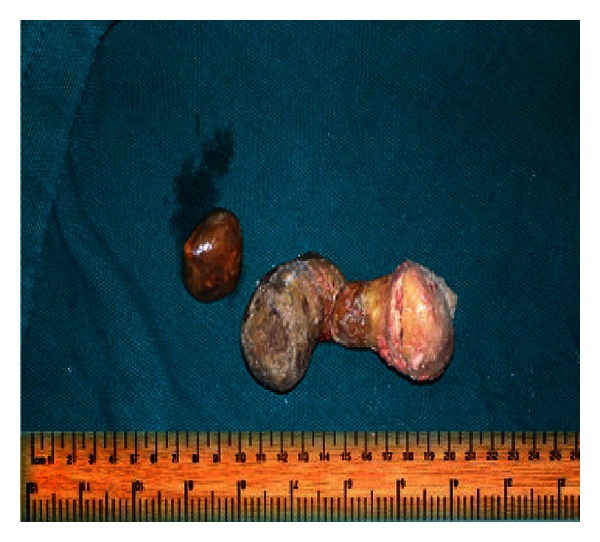
Picture showing two calculi one huge dumbbell shaped calculus and a smaller globular calculus.

*Treatment.* Surgical exploration with removal of calculus was planned. The stones were treated by open transvesical prostatolithotomy, bladder neck incision, and Visual Internal Urethrotomy. Two calculi were found and removed. One was a huge dumbbell calculus of size around 10.2∗3.5∗4.5 cm and the other was a smaller round one of size 2∗1∗0.5 cm (Figure 5). The calculus on biochemical examination showed calcium magnesium ammonium phosphate (triple phosphate). Urinary drainage was achieved through suprapubic catheter (SPC) and a pelvic drain was also placed. A 16 Fr catheter was passed per urethra. Drain was removed on the 2nd postoperative day. Catheter was removed on postoperative day 7. Patient started to pass urine satisfactorily and SPC was removed. The patient had good urinary stream and no retrograde ejaculation and was subsequently discharged.

## 4. Discussion

True prostatic calculi are those calculi that develop in the acini or tissues of the prostate gland. One should never confuse this with prostatic urethral calculi which may be urinary calculi lodged in dilated prostatic urethra or in a pouch of the urethra. Similarly, a calculus which is present in an abscess cavity or diverticulum communicating with the urethra must not be considered a true prostatic calculus [4]. However both may coexist in the same patient. 

Broadly, the discussion can be divided into 2 parts:

- true prostatic calculi,
- prostatic urethral calculi.

### 4.1. True Prostatic Calculi

True prostatic calculi are formed by the deposition of calcareous material on the corpora amylacea [5]. Some reports say that many of them contain constituents found only in urine and not in prostatic secretions. Intraprostatic urinary reflux may be implicated in the formation of prostatic calculi [6]. Prostatic calculi are usually found incidentally during digital rectal examinations, when viewing plain radiographs of the male pelvis in patients who are more than fifty years old, during transrectal ultrasound scan and intraoperatively during transurethral resection of the prostate. They are commonly located at the apical margin of the prostatic adenoma within the line of cleavage between hyperplastic nodule and the surgical capsule [7]. Prostatic calculi may be associated with chronic prostatitis, benign prostatic hyperplasia, or prostate carcinoma [8]. The incidence of prostatic calculi varies from 7% in pathologic specimens, 20% in autopsies, and 30% in radiological studies, to even higher percentages in ultrasound scan examinations [9]. 

Huggins and Bear in 1944 [10] found that the organic components, which compose about 20 percent of the prostatic calculus, include proteins (8%), cholesterol (from 3.7% to 10.6%), and citrate (0.17% to 2.9%). Sutor and Wooley [11] stated that true prostatic calculi comprise solely of calcium phosphate trihydrate (whitlockite) and carbonate.

Based on their composition, prostatic calculi have been classified as primary or endogenous calculi and secondary or exogenous calculi. 

*Primary or endogenous calculi* are formed in acini from prostatic fluid. Endogenous prostatic calculi are usually composed of apatite and whitlockite. They have a compact nucleus with concentric layers of carboapatite and intercalated layers of whitlockite in the periphery. 

*Secondary or exogenous calculi* are formed in the prostatic duct. They are composed initially of crystalline oxalic or uric acid nuclei however, at a later stage they are surrounded or covered by layers of apatite and whitlockite [12]. 

Some authors reported that prostatic calculi produce nonspecific lower urinary tract symptoms (LUTSs) [13]. It has also been stated that there is a clear correlation between age and incidence of prostatic calculi, although the presence of prostatic calculi in younger men is often associated with inflammatory symptoms and prostatitis [14]. König and associates [15] reported that prostatic calculi induce prostatitis and aggravate LUTS. 

The effects of prostatic calculi on LUTS are not very clear however, several explanations have been postulated. Some of these postulates include

1. prostatic calculi probably affect relaxation of the prostatic urethra and hence interfere with the urinary stream. There is a more significant effect on relaxation of the prostatic urethra in periurethral calculi than in scattered stromal calculi [16];
2. another mechanism underlying LUTS is spasm of pelvic floor muscles [17]. Patients with prostatic calculi had more severe irritative symptoms and voiding symptoms in our study. This observation suggests that prostatic calculi induce not only mechanical obstruction but also smooth muscle contraction.

Small asymptomatic prostatic calculi do not require any treatment. The various treatment options available include the following [18].

(a) *Transurethral Removal of the Prostatic Calculi*. This modality of treatment may produce temporary relief but may not necessarily guarantee removal of all the prostatic calculi; therefore, recurrent prostatic calculi formation may be encountered subsequently. Ultrasound guided transurethral removal is a better option particularly for young patients to avoid impairment of sexual activity and to relieve pain or in the older patient who is a poor surgical risk. 

(b) *Suprapubic Removal*. This method of treatment may be used in the presence of a large stone or stones associated with significant prostatic enlargement.

Other available methods of treatment include the following.

(c) *Perineal Prostatotomy or Total Prostatectomy*. Total prostatectomy and bilateral seminal vesiculectomy, although radical, may be considered in the presence of multiple symptomatic calculi and intractable infection.

### 4.2. Urethral Calculus

Urethral calculi are preponderantly found in the prostatic urethra just proximal to the narrow membranous portion. These are either formed in the prostatic urethra or migrated from the upper urinary tract. They are usually small, but numerous cases of giant stones have been reported.

Urethral calculi may be completely asymptomatic or may have few symptoms like perineal or penile pain, frequency, urgency, diminished urinary stream, dribbling and haematuria, or urethral discharge. The diagnosis is based on clinical history and relevant investigations [19].

 Urethral stones are classified as

- *autochthonous or primary* (those formed de novo in the urethra),
- *migrating or secondary* (those formed in the upper urinary tract with secondary downward descent) [20].

*Primary native calculi* are usually small and multiple, and secondary migratory calculi are usually larger. Primary urethral stones are generally composed of magnesium ammonium phosphate (struvite) [21]. They have no nucleus and are of uniform structure. They are formed in the urethra either behind some stricture or within a poorly drained communicating cavity, obstruction, stagnation, infection, and inflammation being the predisposing factors [22].

*Secondary or migratory stones* are usually of calcium oxalate or citrate [23]. They are much more common. They are most often encountered in association with urethral stricture disease or other forms of urethral obstruction. They may be seen in approximately 60% of patients with long-standing urethral stricture disease [24].

Young divides the primary urethral calculi into four groups [25]:

Group I: prostatic urethral calculi associated with prostatitis.

Group II: calculi associated with hypertrophy of the gland.

Group II: calculi that simulate carcinoma.

Group IV: calculi in both the prostatic urethra and the urinary tract.

Swift Joly has classified stones lodged in the posterior urethra into three different categories [26]. 

(a) *Vesico-Urethral Stones*. These stones lie partly in the posterior urethra and partly in the bladder, and therefore lie astride the internal sphincter which is responsible for a constriction, clearly visible on the calculus.

(b) *Urethral Stones*. Stones are localized to the urethra.

(c) *Urethro-Prostatic Stones*. These stones lie partly in the pre-formed cavity in the prostate gland.

In our case, It is a *Joly type “a”* with both bladder and prostatic urethral component resulting in a dumbbell shape and* Young group IV.*

Prostatic urethral calculi occur more frequently in younger men, unlike true prostatic calculi, which are usually seen in men older than 50 years of age [27]. They are exceedingly rare in females because of low incidence of vesicle calculi and shorter urethra [28]. The causative factors can be urethral stricture, stasis, or stagnation, with urinary infection, foreign bodies, debris, bladder neck obstruction, idiopathic factors, lithogenic diathesis, and schistosomiasis [29]. Predisposing factors for in situ development of urethral stones include the presence of urethral diverticulum, urethral stricture, hypospadias, and meatal stenosis [30]. 

The main symptoms are acute urinary retention, frequency, burning sensation in the urethra on urination, burning sensation in the perineum and/or rectum, or stinging in the anus. Other less frequent symptoms were haematuria, dribbling or incontinence, interruption of the urinary stream, and a history of having passed a stone. 

Management of urethral calculi varies according to the site, size, and associated urethral disease. Retrograde manipulation into the urinary bladder followed by litholapaxy or lithotripsy is a suitable procedure for small urethral calculi. Anterior urethral calculi can be removed with instillation of 2% lignocaine jelly, ventral meatotomy, or urethroscopic method. Recently, there has been a trend toward endoscopic removal due to superior equipment and operating skills [31].

Bedir et al. [27] reported the successful treatment of multiple prostatic urethral calculi endoscopically. However, they approached the calculi transurethrally and suprapubically through a suprapubic incision. Brady and Blake of Utah school of medicine reported the successful management of small urethral calculi by transurethral holmium laser ablation. This appears to be an attractive approach in the field of urology [32]. Giant urethral calculi should be treated with open surgery. In urethral stones associated with stricture urethra, urethroplasty is done along with removal of calculus [30]. 

The treatment of choice for an impacted large calculus in the bulbar urethra is perineal urethrotomy with urethroplasty. Similarly, other methods of management of the giant calculus impacted in prostatic urethra include radical prostatectomy, open retro-pubic prostatolithotomy, and endoscopic lithotripsy [33]. Combined giant bladder and prostatic urethral calculi in the same patient is unusual. Giant calculi in prostatic urethra are extremely rare. 

Kim et al. [34] reported the successful removal of a giant prostatic calculus with a concurrent bladder stone transurethrally. The bladder stone was removed first followed by transurethral resection of the prostate with simultaneous removal of the prostatic calculus. This is the first case in our centre and probably the largest giant vesico-prostatic urethral calculus in the English literature. Less than 20 cases of giant prostatic urethral calculi have been reported in the English literature. This case was reported because of the size and dumbbell nature of the stone. Giant urethral calculi are better treated with open surgery. 

## References

[1] Stoller ML, Tanagho EA, McAninch JW (2008). Urinary stone disease. *Smith’s General Urology*.

[2] Koga S, Arakaki Y, Matsuoka M, Ohyama C (1990). Urethral calculi. *British Journal of Urology*.

[3] Ahmed QJ, Gulfam MA, Ghayasuddin (2003). Giant urethral diverticulum with calculi. *Pakistan Journal Of Surgery*.

[4] Venyo AK-G (2012). Prostatic calculi: a review of the literature. *WebmedCentral Urology*.

[5] Menon M, Resnick MI, Walsh PC, Retik AB, Vaughan ED, Wein AJ, Kavoussi LR, Novick AC (2002). Urinary lithiasis: etiology, diagnosis, and medical treatment. *Campbell’s Urology*.

[6] Park HK, Kwak C, Kim SH, Jeong H, Lee SE (2003). The effect of prostatic calculi detected by transrectal ultrasound on the level of serum prostate specific antigen. *Korean Journal of Urology*.

[7] Sant GR, Seidmon EJ, Hanno PM (1994). Prostatic calculi. *Current Urologic Therapy*.

[8] Grayhack JT, McVary KT, Kozlowski JM, Gillenwater JY, Grayhack JT, Howards SS, Mitchell ME (2002). Benign prostatic hyperplasia. *Adults and Paediatrics Urology*.

[9] Kirby RS, Lowe D, Bultitude MI, Shuttleworth KED (1982). Intra-prostatic urinary reflux: an aetiological factor in abacterial prostatitis. *British Journal of Urology*.

[10] Huggins C, Bear RS (1944). Course of prostatic ducts and anatomy: chemical and X-ray diffraction analysis of prostatic calculi. *Journal of Urology*.

[11] Sutor DJ, Wooley SE (1974). The crystalline composition of prostatic calculi. *British Journal of Urology*.

[12] Ramirez CT, Ruiz JA, Gomez AZ (1980). A crystallographic study of prostatic calculi. *Journal of Urology*.

[13] Klimas R, Bennett B, Gardner WA (1985). Prostatic calculi: a review. *Prostate*.

[14] Donnell RF (2005). Antinanobacterial therapy for men with chronic prostatitis/chronic pelvic pain syndrome and prostatic stones. *Current Urology Reports*.

[15] König JE, Senge T, Allhoff EP, König W (2004). Analysis of the inflammatory network in benign prostate hyperplasia and prostate cancer. *Prostate*.

[16] Park SW, Nam JK, Lee SD, Chung MK (2010). Are prostatic calculi independent predictive factors of lower urinary tract symptoms?. *Asian Journal of Andrology*.

[17] Klimas R, Bennett B, Gardner WA (1985). Prostatic calculi: a review. *Prostate*.

[18] Drach GW, Walsh PC, Retik AB, Stamey TA, Vaughan ED (1992). Calculi of the prostate and seminal vesicles in urinary lithiasis: etiology, diagnosis, and medical management. *Campbell’s Urology*.

[19] Deodhar SD, Khope SS (1983). Giant prostatic urethral calculus. (A case report). *Journal of Postgraduate Medicine*.

[20] Shanmugam TV, Dhanapal V, Rajaraman T, Chandmsekar C, Balashanmugam KP (2000). Giant urethral calculi. *Hospital Medicine*.

[21] Usta MF, Baykara M, Erdoğru T, Köksal IT (2005). Idiopathic prostatic giant calculi in a young male patient. *International Urology and Nephrology*.

[22] Lowsley OS, Kirwin JJ (1956). *Clinical Urology*.

[23] Kaplan M, Atakan IH, Kaya E, Aktoz T, Inci O (2006). Giant prostatic urethral calculus associated with urethrocutaneous fistula. *International Journal of Urology*.

[24] McCallum RW, Banner MP, Pollak HM (1992). Lower urinary tract calculi and calcifications. *Clinical Urography*.

[25] Young H (1934). Prostatic urethral calculi. *Journal of Urology*.

[26] Barrett JC (1957). Giant prostatic calculi. *British Journal of Surgery*.

[27] Bedir S, Kilciler M, Akay O, Erdemir F, Avci A, Özgök Y (2005). Endoscopic treatment of multiple prostatic calculi causing urinary retention. *International Journal of Urology*.

[28] Win T (1994). Giant urethral calculus. *Singapore Medical Journal*.

[29] Larkin GL, Weber JE (1996). Giant urethral calculus: a rare cause of acute urinary retention. *Journal of Emergency Medicine*.

[30] Hegele A, Olbert P, Wille S, Heidenreich A, Hofmann R (2002). Giant calculus of the posterior urethra following recurrent penile urethral stricture. *Urologia Internationalis*.

[31] Wollin TA, Singal RK, Whelan T, Dicecco R, Razvi HA, Denstedt JD (1999). Percutaneous suprapubic cystolithotripsy for treatment of large bladder calculi. *Journal of Endourology*.

[32] Walker BR, Hamilton BD (2001). Urethral calculi managed with transurethral Holmium laser ablation. *Journal of Pediatric Surgery*.

[33] Virgili G, Forte F, Sansalone S (2004). Radical prostatectomy as unique chance for huge prostatic stones. *Archivio Italiano di Urologia e Andrologia*.

[34] Kim BH, Kim KJ, Kim SJ (1999). A case of a giant prostatic calculus with bladder stones. *Korean Journal of Urology*.

